# The role of artificial intelligence in paediatric cardiovascular magnetic resonance imaging

**DOI:** 10.1007/s00247-021-05218-1

**Published:** 2021-12-22

**Authors:** Andrew M. Taylor

**Affiliations:** 1 Great Ormond Street Hospital for Children, Zayed Centre for Research, 20 Guildford St., Room 3.7, London, WC1N 1DZ UK; 2 Cardiovascular Imaging, UCL Institute of Cardiovascular Science, London, UK

**Keywords:** Artificial intelligence, Cardiovascular, Children, Heart, Magnetic resonance imaging

## Abstract

Artificial intelligence (AI) offers the potential to change many aspects of paediatric cardiac imaging. At present, there are only a few clinically validated examples of AI applications in this field. This review focuses on the use of AI in paediatric cardiovascular MRI, using examples from paediatric cardiovascular MRI, adult cardiovascular MRI and other radiologic experience.

## Introduction

As with all aspects of imaging, paediatric cardiac imaging is expected to undergo dramatic changes over the next decade in all aspects of the work, driven by the use of artificial intelligence (AI). A recent review of AI in the literature identified five health care areas where AI is expected to have a huge impact [1]: health care systems management, diagnostics, clinical decision-making, patient data and predictive medicine.

**Fig. 1:**
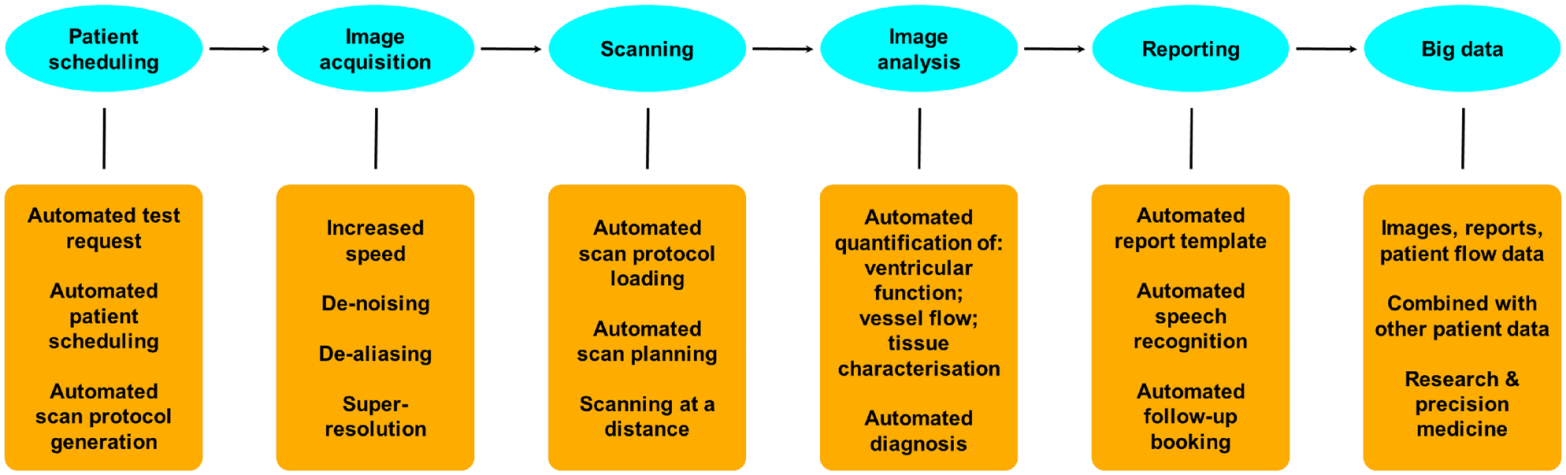
Aspects of the radiologic patient pathway for paediatric cardiovascular MRI where artificial intelligence can play a role

There are few examples of how AI is used in paediatric cardiac imaging. This review presents some of these advances and gives a flavour of the opportunities that AI and other technologies have to offer. I use the patient pathway for a paediatric cardiovascular MRI scan to highlight the areas that can be affected by AI — the processes of ordering, organising, performing and reporting a cardiovascular MR scan and how these data can then be utilised for research and patient benefit (Fig. 1). I also describe some of the ways that AI can impact echocardiography and cardiovascular CT.

Importantly, developments in AI should help move clinical practice from an era of evidence-based medicine to one of intelligence-based medicine [2] and precision patient management, where information from big data is used to create knowledge that is then drilled down to meet the specific needs of the individual patient in real time. Ultimately, this is expected to improve patient outcomes as well as patient and clinician experiences. Ideally, it will also free clinician time from many of the mundane processes that we currently need to carry out.

## How artificial intelligence can affect the patient pathway in cardiovascular magnetic resonance imaging

### Ordering, scheduling and protocolising

The first step in any radiologic procedure is test ordering. The aim is to have the right test (most appropriate test for the clinical question) at the right time (clinical urgency, patient choice) that is efficient (minimising missed appointments and providing the most cost-effective test).

Improving the information on the imaging request form, which is often poor, is a crucial first step to drive all these processes. One recent study found that 80% of imaging requests did not have the relevant clinical information [3]. AI tools can extract clinical information from the clinical electronic patient record (EPR) to improve imaging modality selection [4] and then drive imaging protocolisation to address the relevant clinical questions [3]. One such machine learning tool was able to analyse unstructured text for clinical indications for neurologic MR imaging requests and appropriately protocolise the MR scan with an accuracy of 95% [5]. For cardiovascular MR imaging, prior clinical data and previous imaging protocols (from the individual or patients with similar underlying heart disease) could be used to potentially predict scan times. Furthermore, algorithms could be used to search the EPR to ensure that there are no contraindications to MRI, pre-populating the MR consent form with this information.

Algorithms can also be used to support optimisation of scheduling:

- to define optimal times for appointment bookings [6];
- to predict which patients might not attend [7]; and
- to define optimal follow-up times [8], automatically booking follow-up imaging.

This can improve efficiency — fewer staff calling, texting with appointment reminders, etc. — all of which can lead to an improved patient experience with more timely reminders about dates and times of imaging appointments, fewer missed appointments, improved utilisation of expensive scanner time and appropriate use of staff. Machine learning has also been used to predict wait times and delays on the day of imaging to keep patients and families better informed [9].

### Cardiovascular magnetic resonance sequences

As we know, MRI is an inherently slow process. Hence, algorithms can reduce the amount of data required and support more rapid scanning. This is more so in cardiovascular imaging, where data are acquired not only in three dimensions, but also over time.

Multiple techniques have been developed over the years, including parallel imaging and compressed sensing, to reduce acquisition times for cardiovascular MR sequences. These imaging techniques are prone to signal degradation and aliasing and require prior information, which is often crude at present. AI methods are excellent at discovering patterns and have been used to improve cardiovascular MR image quality for sparsely sampled data. Deep neural networks, in particular convolutional neural networks (CNNs) and recurrent neural networks, have been used to solve machine learning problems, predominately in a supervised way (using known ground-truth data) [10]. Examples include:

- de-noising data (to enable k-space under sampling),
- de-aliasing data (to enable k-space under sampling),
- super-resolution (to use low-resolution data to predict high-resolution detail), and
- reduced errors from field inhomogeneity and eddy currents (more accurate flow imaging).

In paediatric cardiovascular MR, clinical validated machine learning studies have shown that real-time acquisitions for two-dimensional (2-D) cine imaging can be used to reduce scan time from just under 5 min to 18 s with no loss in image quality [11]. This required training in 10 people with congenital heart disease (CHD). For three-dimensional (3-D) whole heart imaging in people with CHD, super-resolution machine learning reconstruction has been shown to reduce scan time from 8 min to 3 min, with similar image quality compared to current methods [12]. Similar integration of AI for rapidly reconstructing four-dimensional (4-D) flow [13] and 3-D late-gadolinium enhancement [14], though not tested in the CHD setting, might also speed cardiovascular MR imaging.

**Fig. 2:**
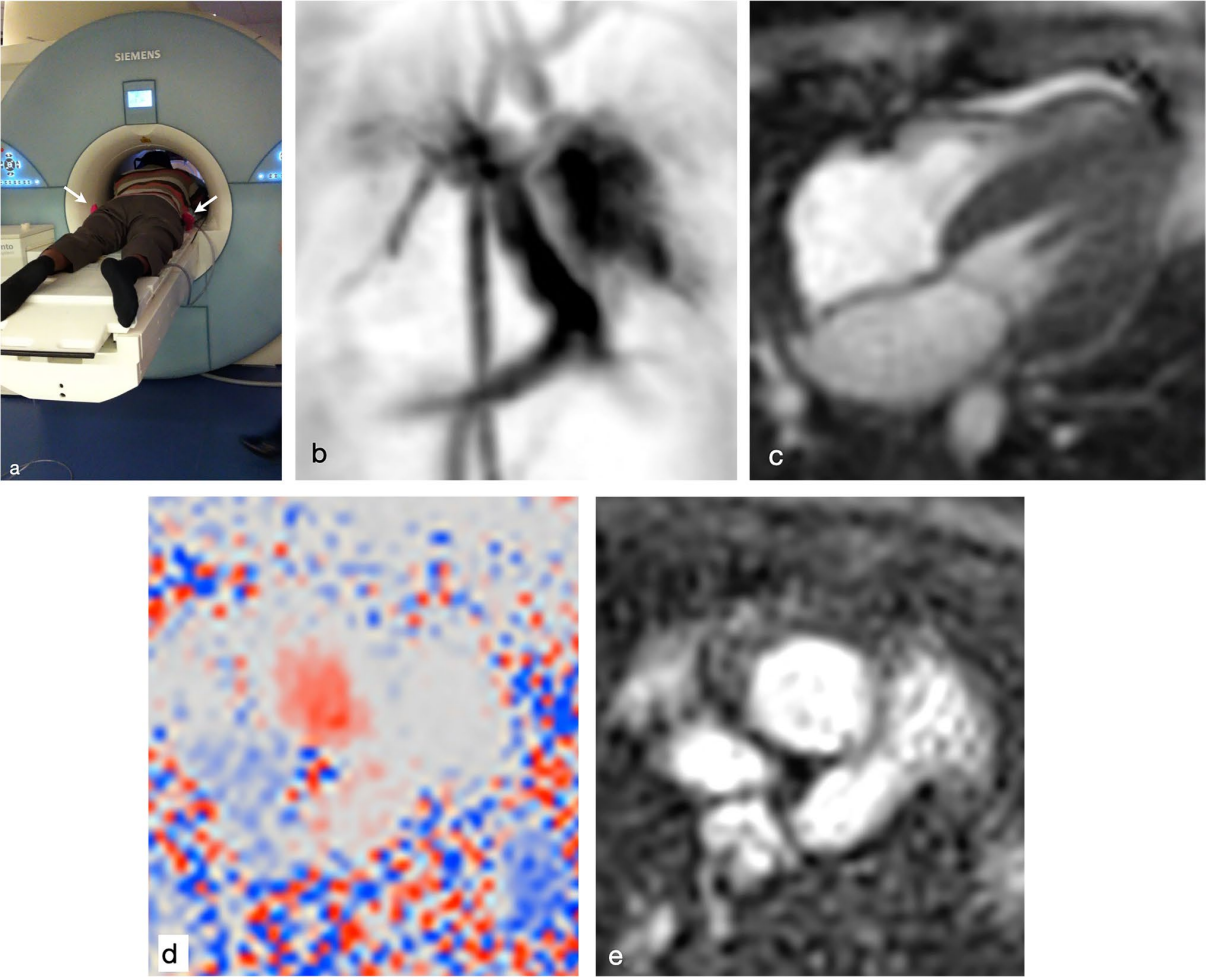
Cardiovascular MR imaging in a 3-year-old girl with pulmonary hypertension without general anaesthetic. **a** Clinical image shows a father comforting his child (*white arrows* show small red socks of child!) during the scan, whilst child watches a video. **b** Anteroposterior time-resolved gadolinium-enhanced MR angiogram of the great vessels in the thorax. **c** Real-time cine imaging of the heart, 4-chamber view. **d, e** Oblique transverse phase-contrast velocity images (phase-contrast image [**d**], magnitude image [**e**]) of the ascending aorta to measure systemic flow

Ultimately all these methods allow for image acquisition to be quicker, reducing the overall scan time by factors of up to 4 (60-min scan now performed in 15 min). For paediatric cardiovascular MR, this allows for reduced need of general anaesthetic for younger children (shorter scans, no need for breath-holding; Fig. 2), better overall image quality from better compliance (again shorter scans, no need for breath-holding) and increased scanner utilisation (more scans performed each session).

### Cardiovascular magnetic resonance scanning

As described, AI might be able to help with cardiovascular MR protocolisation, which would enable protocols to be automatically loaded into the sequence list for the appropriate clinical indication (e.g., basic CHD protocol, heart failure protocol, cardiomyopathy protocol). Furthermore, learning-based algorithms can be applied to enable automated planning for the ventricular imaging planes for imaging the heart, which can take less than 10 s to complete [15]. Another potential way to reduce scan time might be to determine which sequences lead to pertinent clinical information in any given condition, again focusing the imaging protocol.

Similar automated algorithms using CNNs have been used for echocardiography and have included view identification based on validation of more than 8,000 manually segmented echocardiograms, with accurate imaging plane identification (96% for parasternal long axis) [16].

These methods support imaging technicians as they plan cardiovascular MR scans, reducing operator dependence and enhancing the homogeneity of imaging protocols locally and across organisations to enhance data for multicentre studies. This might mean that complex cardiovascular MR scans that require children to travel to distant specialist centres can be carried out to the same standard, utilising the same degree of expertise, using an MR scanner closer to the child’s home — potentially reducing waiting times for scans and reducing travel times for patients and families.

### Cardiovascular magnetic resonance analysis

#### Imaging segmentation/data analysis

As we continue to reduce the length of cardiovascular MR scans, benefiting patients, and increasing the number of scans we can perform, we remove one of the bottlenecks for cardiovascular MR waiting lists. However, the burden now shifts to the reporting environment. Currently, complex cardiovascular MR scans can take more than 60 min to report, with manual segmentation of ventricular volumes (dreaded circle drawing), flow post-processing (including 4-D flow), 3-D anatomy segmentation and tissue characterisation, all requiring significant cardiac imager time to analyse. It is therefore essential that these mundane tasks become more automated, to reduce reporting times and prevent a second bottleneck (no waiting for cardiovascular MR appointments because scans can be done quickly, but referrers now waiting for scan reports — the *new bottleneck*). Furthermore, algorithms that could compare a patient’s studies to determine changes in volumes, flow, function and other imaging characteristics would be hugely useful, saving enormous amounts of time comparing studies and providing a trend for patient disease progression to track management (e.g., response to drug treatments, response to surgery or interventional cardiac catheterisation, or watchful waiting as part of routine surveillance).

Fortunately, a whole array of AI algorithms — machine and deep learning — are being developed to assist in data analysis, including automated quantification of ventricular volume, automated flow analysis, automated segmentation for tissue characterisation and automated cardiovascular MR tissues fingerprinting. They are described in the next paragraphs.

##### Automated quantification of ventricular volume

CNNs using multi-vendor left ventricular (LV) data have shown excellent correlation (r^2^ ≥ 0.98) and agreement with measurement obtained by clinical experts [17]. Similarly good agreement has been found for LV volume and function assessment using a deep-learning-based algorithm for fully automated quantification [18]. Measurements of LV, left atrial (LA), right ventricular (RV) and right atrial (RA) volumes have also been shown to be accurate using a fully convoluted network with a large-scale annotated dataset, comparable to human interobserver variability using United Kingdom biobank data [19]. Deep learning algorithms can now be integrated into frameworks for cardiovascular MR cardiac functional assessment, which can provide a degree of quality control. One such framework consists of pre-analysis deep learning image quality control, followed by a deep learning algorithm for biventricular segmentation in long-axis and short-axis views, myocardial feature-tracking, and a post-analysis quality control to detect erroneous results [20]. The framework achieved good agreement with no clinical oversight, though some RV measures were less well performed by the framework than manual assessment. Importantly, deep learning ventricular segmentation of both the LV and RV has now been shown to be useable in CHD, though accuracy for RV measurements was worse than for LV measurements [21].

##### Automated flow analysis

Although flow segmentation is relatively automated for the majority of cardiovascular MR post-processing tools, further automation to analyse images with limited or no operator input would be useful. A recent study looking at ascending aortic flow measurements at the aortic valve level has shown that accurate machine learning segmentation was uniformly successful, requiring no human intervention, with very high correlation (r = 0.99) between manual and algorithm segmentation and measurement in an external validation cohort [22].

##### Automated segmentation for tissue characterisation

Late-gadolinium enhancement can be used to guide clinical treatment, and clinical implementation of this information requires reproducible and reliable segmentation of the infarcted regions [23]. One automated method using deep CNNs to automatically quantify LV mass and scar volume in people with hypertrophic cardiomyopathy showed potential, with good agreement with manual segmentation [24].

##### Automated cardiovascular MR tissues fingerprinting

Combining T1, T2, extracellular volume and late gadolinium enhancement data to create multi-parametric myocardial tissue characterisation has been demonstrated using pattern recognition algorithms to match the sampled signal to a pre-defined dictionary of predicted signal evolutions [25]. Such automated tissue characterisation could then be useful for automated diagnosis of cardiomyopathies and acute myocarditis and for the monitoring of cardiac transplant rejection.

#### Automated diagnosis

Automated diagnosis in CHD is not straightforward. One attempt has been to use an atlas-based computer-aided approach, which exploits similarity measures between the atlas and target images for normal cardiac anatomy and people with transposition of the great arteries following either the atrial or arterial switch operation. The method was validated using annotated images and subsequently showed a diagnostic accuracy of 97.3% when evaluated on a set of 60 whole heart MR images [26]. Deep learning algorithms have been shown to make the diagnosis of cardiac amyloid from cine cardiovascular MR and late-gadolinium images [27]. Such algorithms might then be able to differentiate between cardiomyopathies not only in adults, but also in children.

Considerable work is required to build and validate algorithms that can automatically diagnose CHD from 3-D images. However, as a first step, algorithms that can distinguish normal from abnormal cardiovascular MR images might be useful to ensure that complex CHD anomalies, which are rare and hence not seen often in routine clinical practice, are not missed, even if the exact diagnosis is not made, and that referral for expert opinion is carried out.

### Cardiovascular magnetic resonance report generation

Algorithms could be used to generate a report template based on the clinical referral and the clinical patient information from the EPR. This information could also be used to search local and international clinical guidelines and research publications so that concurrent literature related to the case is instantaneously available at the time of reporting. For example, when reporting a case of Turner syndrome, the real-time availability of the current aortic dimensions from the latest consensus paper to guide clinical decisions would be hugely valuable.

Report generation can clearly be supported by automated speech recognition that either gathers data in a structured form or extracts data using natural language processing tools [28] to ensure that data are available for big data sets. The data from the radiologic report could also be used to automatically inform the referring clinician and used to drive follow-up recommendations — e.g., “Follow-up assessment for right ventricular volume assessment recommended in 2 years” automatically generates a cardiovascular MR appointment for the patient at this time point [29]. Furthermore, AI could be leveraged to generate targeted reports, specific for the referring cardiologist, the patient and the family, or the non-cardiologist, to ensure that the most pertinent information gets to the right person in the right language for them to understand and affect patient care.

### Provision of data for research and precision medicine

**Fig. 3:**
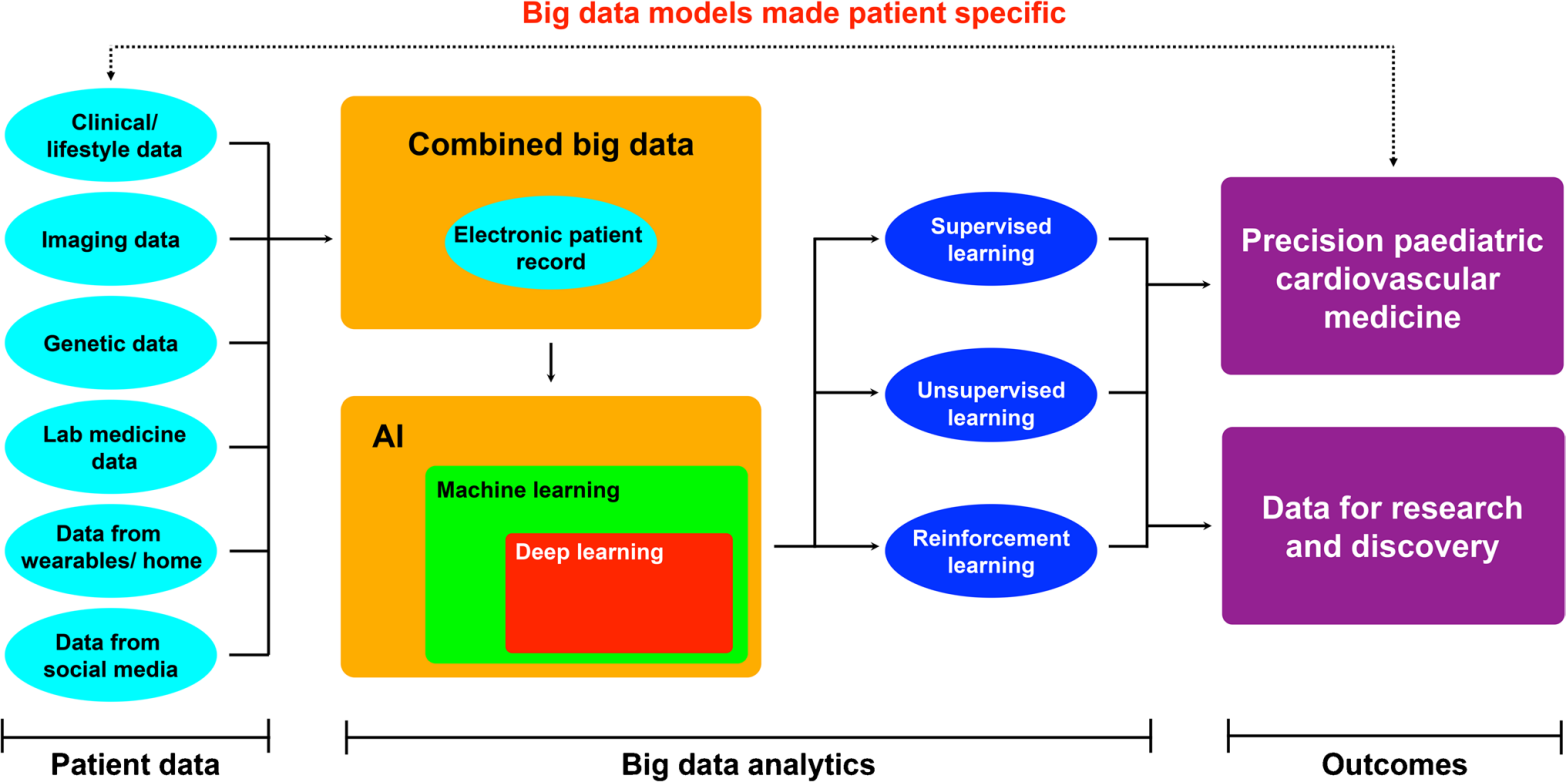
Integration of patient information from multiple sources brought together in a single electronic patient record. These data, and data from thousands of other patients, are accessed using artificial intelligence (AI) and learning algorithms to create new understanding for patient populations. These big data can then be accessed for the individual patient, using AI and supervised, unsupervised and reinforcement learning to provide precision care in a feedback cycle, creating a virtuous cycle of patient data and care

The integration of data from multiple sources — clinical, imaging (radiology, digital pathology, photographs), genomic, proteomics and laboratory medicine — can be used to create vast databases that can be explored by AI algorithms (Fig. 3). After these databases have been explored and validated, information can then be drilled down back to individual patients to drive precision medicine for each person based on their clinical history, imaging, laboratory findings and genetics, combined with the latest outcomes gathered in real time.

A few studies have used machine learning to combine cardiac imaging with other investigative and clinical features to predict prognosis. In one study, cardiovascular MR imaging data were combined with clinical, haemodynamic and functional markers in adults with pulmonary hypertension [30]. A machine learning survival model that included the cardiac motion parameters derived from cardiovascular MR was shown to have incremental prognostic power when compared with conventional parameters [30].

In a second study, measures of cardiac dimensions and function were derived from cardiovascular MR images of people with tetralogy of Fallot using automated deep learning analysis, which was combined with established clinical parameters and electrocardiogram (ECG) markers [31]. The deep learning models were trained on raw Digital Imaging and Communications in Medicine (DICOM) data at a single centre, then applied to a national cohort dataset to predict prognosis for adults with CHD.

## Summary of artificial intelligence in other areas of cardiovascular imaging

Authors Xu et al. [32] gave an excellent summary of the AI developments in echocardiography and cardiovascular CT and the tools that have been developed.

### Echocardiography

Artificial intelligence lends itself to all areas of echocardiography with automated analysis of chambers, ejection fraction and strain [16], valvular assessment [33] and aortic dimensions [34], with a move toward automatic diagnosis that has been shown in several areas, including the diagnosis of constrictive versus restrictive cardiomyopathy [35] and hypertrophic cardiomyopathy versus the athlete’s heart [36].

### Cardiovascular computed tomography

At present, the majority of algorithms that have been developed for cardiovascular CT have focused on the assessment of ischaemic heart disease with algorithms to support anatomical diagnosis, coronary artery plaque quantification, coronary blood flow and myocardial perfusion.

A combination of clinical and cardiovascular CT parameters using a machine learning framework has been shown to better predict 5-year mortality than conventional measures using the Framingham risk score [37]. Coronary artery blood flow — fractional flow reserve — can be calculated using computational fluid dynamics modelling [38] with excellent accuracy that has been demonstrated in several prospective multicentre studies.

Such algorithms might prove useful in the analysis of small stenosed vessels in CHD cardiovascular CT, and methods for automated CHD diagnosis developed for 3-D cardiovascular MR images should be useable in cardiovascular CT when they become available.

## Conclusion

There are few concrete examples of the use of clinically validated AI in paediatric cardiac imaging. However, there is clearly huge potential to improve the experiences of children and clinicians in health care, using AI to drive precision patient medicine and disease discovery by combining siloed data sets from the whole patient pathway. Ultimately, this is expected to build an era of intelligence-based medicine that should enable more proactive, as opposed to reactive, care over time to support preventative medicine and early disease detection.

Much work remains to validate AI technologies in paediatric practice and to address some of these issues:

- changing the way care is delivered/clinical teams are managed;
- building good-quality (excellent data in) large data sets, which can be difficult in rare diseases;
- moving from single-centre validation to universal applicability;
- understanding data issues of confidentiality (patients and families in the paediatric setting), ownership (who owns health care data?), and value (what is health care data worth?);
- importance of regulation for ethical use of data, but not over-regulation that stifles crucial innovation;
- importance of understanding the role of decision support tools/models of care, and where AI information does not make sense; and
- the need for connectivity to link disparate data sets coming from the home, primary care and the hospital settings, and linking clinical teams, patients and families, and data scientists and engineers together.

If we can reduce the amount of mundane work we have to do, speed data acquisition and automate data analysis and diagnosis, whilst having all the relevant information at our fingertips to plan patient care based on the most recent guidelines, providing information that can then be used to drive the next person’s care in a virtuous cycle, AI will ideally give us more time to think and talk to our patients and their families, paradoxically increasing the human touch that is so vital for patient care.
